# Fractionation and characterization of lignin streams from unique high-lignin content endocarp feedstocks

**DOI:** 10.1186/s13068-018-1305-7

**Published:** 2018-11-08

**Authors:** Wenqi Li, Kirtley Amos, Mi Li, Yunqiao Pu, Seth Debolt, Arthur J. Ragauskas, Jian Shi

**Affiliations:** 1 Biosystems and Agricultural Engineering, University of Kentucky, Lexington, KY 40506 USA; 2 Department of Horticulture, University of Kentucky, Lexington, KY 40506 USA; 3 Joint Institute of Biological Science, Biosciences Division, Oak Ridge National Laboratory, Oak Ridge, TN 37831 USA; 4 Department of Chemical and Biomolecular Engineering, University of Tennessee, Knoxville, TN 37996 USA; 5 Department of Forestry, Wildlife, and Fisheries, Center for Renewable Carbon, University of Tennessee Institute of Agriculture, Knoxville, TN 37996 USA

**Keywords:** Endocarp, Deep eutectic solvent, Pretreatment, Lignin, Biofuel, Biorefinery

## Abstract

**Background:**

Lignin is a promising source of building blocks for upgrading to valuable aromatic chemicals and materials. Endocarp biomass represents a non-edible crop residue in an existing agricultural setting which cannot be used as animal feed nor soil amendment. With significantly higher lignin content and bulk energy density, endocarps have significant advantages to be converted into both biofuel and bioproducts as compared to other biomass resources. Deep eutectic solvent (DES) is highly effective in fractionating lignin from a variety of biomass feedstocks with high yield and purity while at lower cost comparing to certain ionic liquids.

**Results:**

In the present study, the structural and compositional features of peach and walnut endocarp cells were characterized. Compared to typical woody and herbaceous biomass, endocarp biomass exhibits significantly higher bulk density and hardness due to its high cellular density. The sugar yields of DES (1:2 choline chloride: lactic acid) pretreated peach pit (*Prunus persica*) and walnut shell (*Juglans nigra*) were determined and the impacts of DES pretreatment on the physical and chemical properties of extracted lignin were characterized. Enzymatic saccharification of DES pretreated walnut and peach endocarps gave high glucose yields (over 90%); meanwhile, compared with dilute acid and alkaline pretreatment, DES pretreatment led to significantly higher lignin removal (64.3% and 70.2% for walnut and peach endocarps, respectively). The molecular weights of the extracted lignin from DES pretreated endocarp biomass were significantly reduced. ^1^H–^13^C HSQC NMR results demonstrate that the native endocarp lignins were SGH type lignins with dominant G-unit (86.7% and 80.5% for walnut and peach endocarps lignins, respectively). DES pretreatment decreased the S and H-unit while led to an increase in condensed G-units, which may contribute to a higher thermal stability of the isolated lignin. Nearly all β-*O*-4′ and a large portion of β-5′ linkages were removed during DES pretreatment.

**Conclusions:**

The high lignin content endocarps have unique cell wall characteristics when compared to the other lignocellulosic biomass feedstocks. DES pretreatment was highly effective in fractionating high lignin content endocarps to produce both sugar and lignin streams while the DES extracted lignins underwent significant changes in SGH ratio, interunit linkages, and molecular sizes.

**Electronic supplementary material:**

The online version of this article (10.1186/s13068-018-1305-7) contains supplementary material, which is available to authorized users.

## Background

Almost one quarter of the world’s population has unmet basic energy needs and the unprecedented green-house gases emission is causing global climate change [1]. These grand challenges have promoted the development of renewable fuels and materials as alternatives to the petroleum based fuels and chemicals [2]. Lignocellulosic biomass is a complex conglomerate of different biopolymers (such as polysaccharides, lignin and protein). From a biorefinery perspective, polysaccharides provide a sugar stream for biofuel fermentation; while the value of lignin has not been fully tapped, the aromatic nature of lignin makes it a potential source of chemicals and materials [3]. Biofuels community are now increasingly interested in fractionating and upgrading lignin to building blocks for high value-added chemicals and materials. Lignin based co-products will greatly enhance the economic viability of a biorefinery [4].

**Fig. 1:**
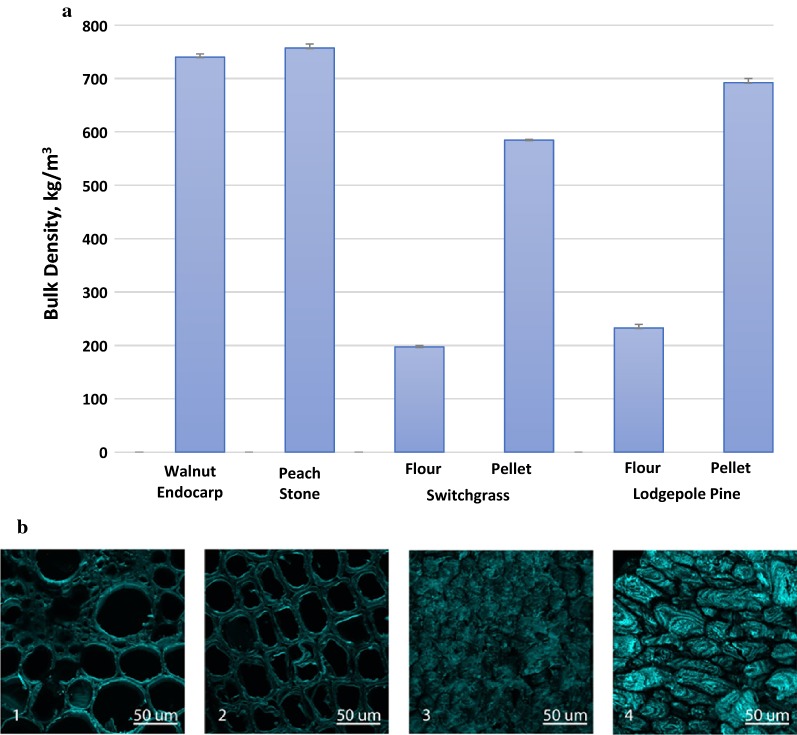
**a** Bulk density of endocarp biomass in flour form in comparison with switchgrass and lodge pole pine in flour and pellet forms [7]; **b** confocal microscopographs of Calcoflour White stained raw biomass comparing (1) switchgrass stem (1st internode), (2) pine stem (heartwood), (3) walnut endocarp, and (4) peach endocarp

As an existing underutilized feedstock from horticultural fruit crops, endocarp is the hardened inedible portion of the fruit which encases the seed and is discarded. Based on the year 2015 USDA Fruit and Tree Nuts Yearbook, the estimated overall annual yield of endocarp biomass from US processing plants reached nearly 1 million dry tons, which breaks down to almonds: 517.0, walnut: 120.0, peach: 63.6, pistachios: 35.0, olives: 22.7, cherries: 16.5, apricots: 2.2, prunes and plums: 0.13, in 1000 dry tons [5]. The hardened drupe endocarp represents the highest lignin content of any biomass source produced in appreciable amounts, up to 50% weight percent [6]. The lignin rich biomass can be a preferable feedstock for biorefinery to produce both biofuel and value-added chemicals and materials. In addition to provide plenty of lignin resources as feedstock, the bulk densities of ground endocarp biomass (i.e., walnut and peach) are 3–4 times higher than the other bioenergy feedstocks such as switchgrass, poplar and pine, as shown in Fig. 1a. The bulk and energy density of the feedstock plays a significant role in the overall energy and cost balance of a biorefinery [7]. A biomass feedstock with high bulk and energy density is more efficient to convert into a biofuel than one with a lower bulk and energy density due to the relatively low energy requirements for transportation, storage, and distribution of the feedstock from the field to the biorefinery gate [8]. Furthermore, the endocarp biomass can be readily collected from the well-established fruit and tree nut processing industry, representing a significant advantage in terms of feedstock supply chain stability and logistics.

To introduce a better use of lignocellulosic biomass to biofuels and lignin-based co-products, it is necessary to find a way to fractionate lignin and cellulose from the feedstock at high efficacy and low cost. Several pretreatment techniques have been studied over the years, with hot water, dilute acid, alkali, and ionic liquid (IL) being the most extensively investigated [9]. Hot water pretreatment is effective in releasing hemicellulose sugars and improving cellulose digestibility to glucose by cellulolytic enzymes [10, 11]. Compared with hot water pretreatment, dilute acid pretreatment can process a wider range of biomass types and achieve higher monomeric sugar yields [12, 13]. In contrast, pretreatment can also be effective at higher pH levels by adding reagents such as lime, calcium carbonate, green liquor, potassium hydroxide, and sodium hydroxide, all of which tend to remove a high fraction of the lignin while removing much less hemicellulose than for dilute acids [14]. During an alkali pretreatment, the ester bonds cross-linking between lignin and xylan are typically cleaved, thus increasing the accessibility of cellulose and hemicellulose enriched fractions to enzymatic digestion [15–17]. However, the subsequent hydrolysate conditioning to remove inhibitors, the higher cost for reaction vessels and solvents, and the waste stream treatment can add extra cost to the overall process, and thus seriously curb the commercialization of these traditional pretreatment techniques [18–20].

IL is named to reflect the unique property of a group of molten salts with melting points below 100 °C. The near infinite possible combinations of cations and anions to form ILs provide opportunities to fine tune their property and functionality, therefore ILs are often called “designer solvents” [21, 22]. Recent advances in deep eutectic solvents (DES) provided a new way for biomass fractionation and lignin extraction application. DES is a mixture of two or more chemicals acting as either hydrogen-bond donors (HBD) or hydrogen-bond acceptors (HBA) [23]. Many DESs share similar properties as ILs towards dissolving lignin from plant materials while costs much less than many ILs due to low precursor price, simple synthesis and better recyclability [24]. The interactions between HBD and HBA of the DES provide a dual acid–base catalysis mechanism which will facilitate controlled cleavage of labile ether linkages among phenylpropane units and thus lead to lignin depolymerization [25]. This chemistry can be tuned by selecting suitable HBD and HBA which will generate a low molecular weight lignin product while maintaining most of the properties and activity of native lignin [26]. A few studies have reported applications of DES for extracting lignin from grass and agricultural residues [27, 28]. Recent studies also investigated deploying this new lignin extraction method to both hardwood and softwood, and characterized the resulting DES extracted lignin product [26, 29]. The resulting lignin product has several distinctive characteristics: high purity, lower and narrowly distributed molecular weight compared to mill wood lignin, and the highly cleaved ether linkages [26]. In addition, DES solvents can be recovered and reused by removing the ethanol and water added for lignin precipitation [26]. After 3 pretreatment cycles, the recovered choline chloride:ethylene glycol (ChCl:EG) retained high lignin removal rate as compared to the pristine DES solvent [30].

The efficacy of a pretreatment method largely depends on the selection of biomass feedstock; at the same time, the selection of a pretreatment technology greatly influences biomass decomposition and sugar release [31, 32]. However, very limited information is available on fractionating endocarp biomass, especially using DES. Therefore, to demonstrate the effect of DES pretreatment on endocarp biomass for production of both sugar stream and high-quality lignin, a choline chloride and lactic acid DES solvent was applied to pretreat peach and walnut endocarp biomass. Sugar yields of pretreated solids were recorded and the mass balances for DES pretreatment and enzymatic hydrolysis for both endocarps were determined. Fractionated lignin streams were characterized using thermogravimetric, spectrometric, gel permeation chromatography and NMR analyses. This study provides insights on possible ways to fractionate and upgrade the underutilized endocarp feedstocks for biofuels and products.

## Results and discussion

### Structural and compositional analysis of raw endocarps

Compared to other biomass feedstocks, endocarp biomass exhibits distinctive compositional and physical properties, such as high lignin content, high bulk density, and hardness. It is not clear how these properties correlate to the plant cell wall structure and its recalcitrance; such knowledge will guide the selection of a suitable pretreatment technology. In comparison with well-known biomass feedstock, such as switchgrass and pine, the structural property of walnut and peach endocarp feedstocks was examined using scanning electron microscopy (SEM) and confocal laser scanning microscopy (CLSM). As can be seen from SEM images in Additional file 1: Figure S1, the switchgrass and pine wood samples retained fibrous nature, while the edges were partially disrupted due to mechanical cutting and grinding. In contract, the walnut and peach endocarps samples showed particulate nature and smaller sample sizes, indicating that the endocarps are brittle. CLSM reveals a three-dimensional cell wall structure of endocarp and biomass samples by capturing multiple two-dimensional images at different depths. Calcofluor white was used to stain cellulose and chitin and is commonly used in plant biology to stain cell walls [33]. Figure 1b compared walnut and peach endocarps to switchgrass and pine wood via CLSM. It is evident that peach and walnut endocarps exhibit a smaller cellular shape and an increase in cellular density when compared to switchgrass and pine wood samples.

**Fig. 2:**
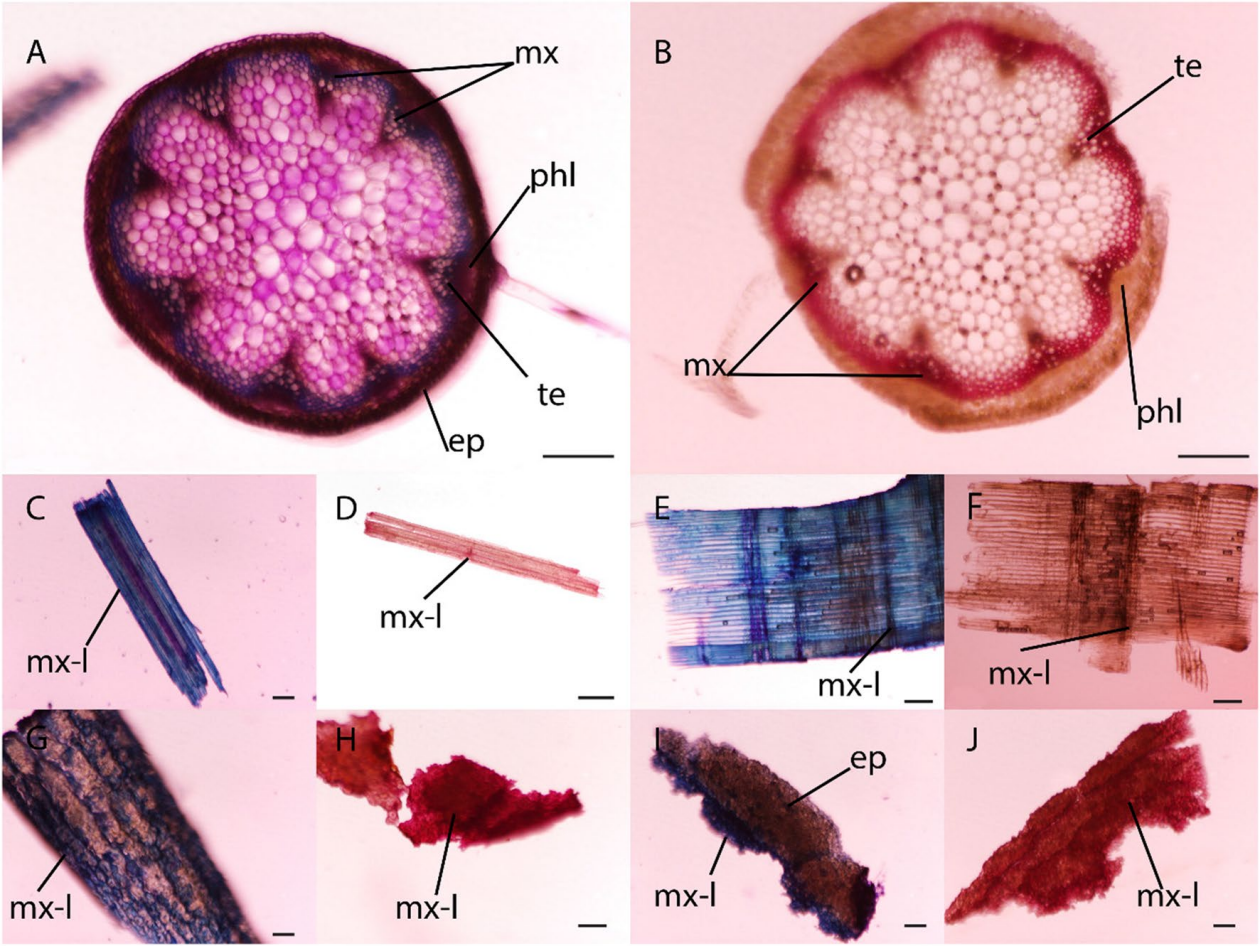
Histochemical evaluation of the lignified nature of peach and walnut endocarps. Evaluation of endocarp used the anatomically characterized Arabidopsis stem section from the lower stem (before first leaf) that have primary and secondary cell walls developed. *mx* metaxylem, *phl* phloem, *te* tracheary elements, *ep* epidermis (note that the cortex is not well defined and grouped with the epidermis),* mx-l* metaxylem-like staining. **A** Toluidine blue staining of transverse cross sections of Arabidopsis stem tissue revealed clear demarcation of the metaxylem in blue, which was also reflected as being highly lignified in the phloroglucinol (**B**) stained stem cross section due to its cherry red color. The switchgrass and pine shavings stained with toluidine blue (**C**, **E**) display a metaxylem-like tissue at a very similar capacity when compared to peach and walnut (**G**, **I**). Phloroglucinol staining displays a marked increase in lignin abundance within the peach and walnut endocarps (**H**, **J**) when compared to the switchgrass and pine samples (**D**, **F**). Scale Bars (100 µM = **C**, **G**, **I**, **J**/200 µM = **A**, **B**, **E**, **F**, **H**/500 µM = **D**). Magnification (×2 = **D**/×4 = **E**, **F**/×5 = **A**, **B**, **H**/×6.3 = **C**, **G**, **I**, **J**)

Additionally, light microscopy was employed to determine the location and distribution of lignin within all four biomass types, as shown in Fig. 2. The anatomy of a transverse cross section of Arabidopsis stem tissue (beneath the first leaf) is well characterized and multiple metachromatic and monochromatic dyes have been used to spatially illustrate composition [33]. Therefore, the use of well characterized stains when applied to Arabidopsis can be used as a proxy when staining other tissues, such as endocarps. Figure 2A, B depicts primary and secondary cell wall staining of Arabidopsis through the use of two dyes (1) Toluidine Blue and (2) Phloroglucinol. Toluidine blue is a metachromatic cationic dye that binds to negatively charged compounds with a primary use in detecting pectin and lignin [34, 35]. Toluidine blue will react with carboxylated polysaccharides and turn pinkish purple, greenish blue or bright blue with poly-aromatic substances, and purplish or greenish blue with nucleic acids [33]. Figure 2A shows a blue staining in the metaxylem that is consistent with proper lignin deposition. Figure 2C, E, G, I show a similar blue color after Toluidine blue staining, in what we called, metaxylem-like tissue. The relative abundance of metaxylem-like tissues within switch grass and pine (C, E) seemed comparable to those within peach and walnut biomass (G and I). To further analyze lignin deposition within tissues, a phloroglucinol stain can be employed [36]. Although it is not a true lignin stain, such that it only stains cinnamaldehyde groups, it is the most common stain for lignin determination. This stain yields a cherry red color in the metaxylem where these groups are present [37]. Figure 2B shows a cherry red color in the metaxylem due to the presence of lignin and, therefore, lignin is abundantly present in the endocarp of peach and walnut (H and J) when compared to switch grass and pine (D and F).

The compositions of walnut and peach endocarps before and after DES pretreatment are shown in Table 1. Unlike other plant materials, lignin contents were much higher, 45.4% and 45.0% for walnut and peach endocarp, respectively. The xylan contents for both endocarps were about 15%, comparable to other biomass feedstock, however, the cellulose contents were lower than woody and herbaceous biomass [38, 39]. Only trace amount (< 1%) of galactan, mannan, and arabian were detected in endocarps, indicating that the plants inherit the hardwood characteristics of peach and walnut trees. Glucan and xylan in total accounting about 30–35%, despite low, still represent a substantial portion of the endocarp biomass. It is worth noting that the extractives were low, however, about 10.2% of walnut and 16.9% of peach endocarp contents were not accounted as lignin or sugars. Those are likely pectins that glue the endocarp cell wall together.

**Table 1:** Composition of raw endocarps and DES pretreated solids

	Peach (%)		Walnut (%)	
	Raw	DES	Raw	DES
Solid recovery	–	34.2 ± 2.8	–	40.5 ± 3.2
Glucan	17.6 ± 2.0	47.1 ± 0.9	20.9 ± 1.1	47.4 ± 3.7
Xylan	15.3 ± 0.0	4.7 ± 0.2	14.9 ± 0.6	4.2 ± 0.6
Galactan	0.4 ± 0.0	ND	0.9 ± 0.0	ND
Arabinan	0.5 ± 0.0	ND	0.4 ± 0.0	ND
Lignin	45.0 ± 3.6	39.2 ± 1.4	45.4 ± 1.2	40.0 ± 2.7
Extractives	2.8 ± 0.1	ND	7.1 ± 0.2	ND
Ash	1.2± 0.3	ND	0.6 ± 0.0	ND

Data represent means and errors are standard deviation from the mean of three independent replicates

*ND* not determined

### Effect of DES pretreatment on lignin extraction efficiency and enzymatic saccharification

**Fig. 3:**
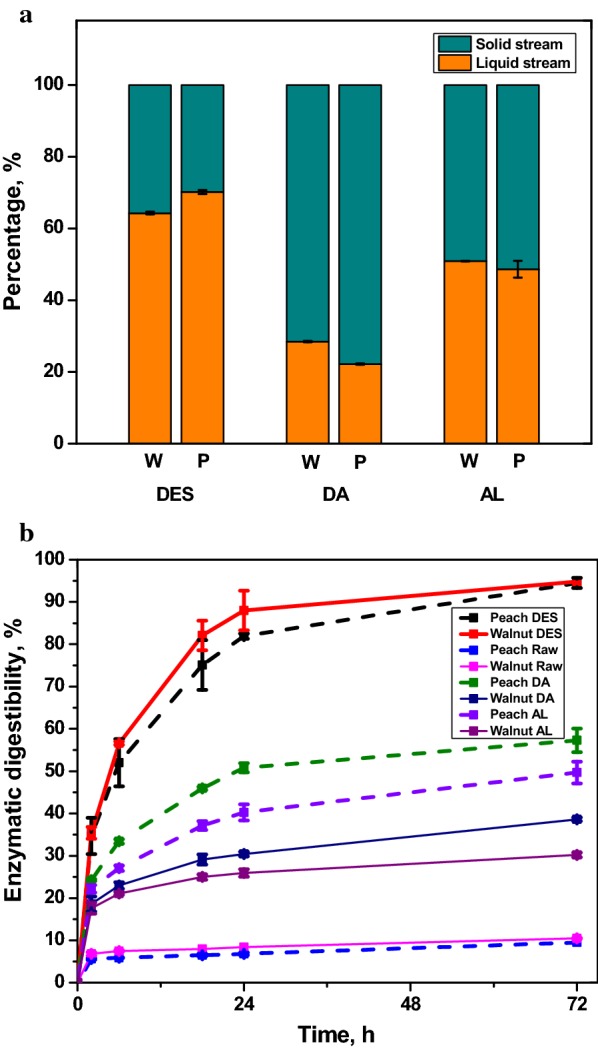
**a** Effects of three pretreatment methods using deep eutectic solvent (DES), dilute acid (DA), and alkaline (AL) on lignin fractionation into pretreatment liquid and solid residue streams for peach (P) and walnut (W) endocarps; **b** enzymatic hydrolysis profiles of untreated, DES, DA, and AL pretreated peach and walnut endocarps (data points represent means and error bars are standard deviation from the mean of three independent replicates)

Impact of DES pretreatment on the compositions of pretreated biomass is summarized in Table 1. Compared with the raw endocarps, the DES pretreated walnut endocarp had higher glucan content (47.4%) but lower xylan (4.2%) and lignin (40.0%) contents. Similar trend was observed for DES pretreated peach endocarp (47.1% of glucan, 4.7% of xylan and 39.2% of lignin). The purity of DES pretreated lignin can achieve up to 92.1% and 93.7% for the extracted walnut and peach lignin, respectively. In addition, the DES pretreatment exhibited a more efficient lignin solubility than the alkaline and dilute acid pretreatment in the present study. As shown in Fig. 3a, lignin removal for DES pretreated walnut and peach endocarp were 64.3% and 70.2%, respectively, which were significantly higher than that of the dilute acid pretreatment (28.5% and 22.2% for walnut and peach endocarp, respectively) and the alkaline pretreatment (50.9% and 48.7% for walnut and peach endocarp, respectively).

Several other pretreatment technologies were also reported to promote sugar release from enzymatic hydrolysis of endocarp biomass. By sequential use of diluted H_2_SO_4_ and NaOH pretreatment, 88% of hemicellulose and 64.4% of lignin within buriti (*Mauritia flexuosa*) endocarp were removed, respectively, which lead to a glucose yield of 86% [40]. Steam-explosion pretreated olive stones (200–236 °C for 2–4 min) contributed to an 87.7% glucose yield in first 8 h of saccharification [41]. It is well known that low pH pretreatment technologies contribute more to the hydrolysis of hemicellulose, while high pH value strategies are mainly directed at lignin but leave a large portion of hemicellulose in the pretreated solid [42, 43]. Our results suggest that DES pretreatment is highly effective in lignin removal, which agrees with previous reports on choline chloride/lactic acid (ChCl:Lac) based DES pretreatment of poplar and Douglas fir [26], rice straw [28], and willow [29]. Results from this work along with previous studies demonstrate that DES pretreatment was a feedstock agnostic pretreatment method capable of fractionating lignin from a variety of biomass feedstocks, including endocarp biomass, with high lignin recovery and purity.

The effect of DES pretreatment on endocarp biomass was further evaluated by enzymatic saccharification of the pretreated endocarp solids, as shown in Fig. 3b. For untreated endocarps, low sugar conversion rates of 10.5% and 9.5% were achieved with saccharification of walnut and peach endocarp, respectively. The DES pretreated endocarps solids showed significantly higher 72-h saccharification sugar conversion rates of 94.8 and 94.5% for walnut and peach endocarps, respectively. In comparison, both dilute acid and alkaline pretreatment exhibited significantly lower sugar conversion rates, which are 38.6 and 57.3% for dilute acid pretreated walnut and peach endocarps, 30.2 and 49.7% for alkaline pretreated walnut and peach endocarps. Results indicate that DES pretreatment can greatly enhance enzymatic saccharification of both endocarps due to the substantial removal of xylan and lignin as discussed earlier. SEM images of the DES pretreated endocarps and the extracted lignin further illustrated the structural changes (Additional file 1: Figure S2). As compared to the intact and highly ordered structure of untreated endocarp samples, the pretreated samples exhibited deeply etched surfaces and reduced sample sizes, which can be attributed to the deconstructive impact of DES solvent due to the removal and re-arrangement of lignin in addition to dissolve of xylan. The extracted lignin appeared as amorphous globous reflecting the dissolution and re-precipitation of lignin during the pretreatment and ethanol–water precipitation and washing process. SEM results provide further evidence that DES pretreatment is effective in enhancing enzymatic hydrolysis by disrupting cell structure and making cellulose more accessible to enzymes.

**Fig. 4:**
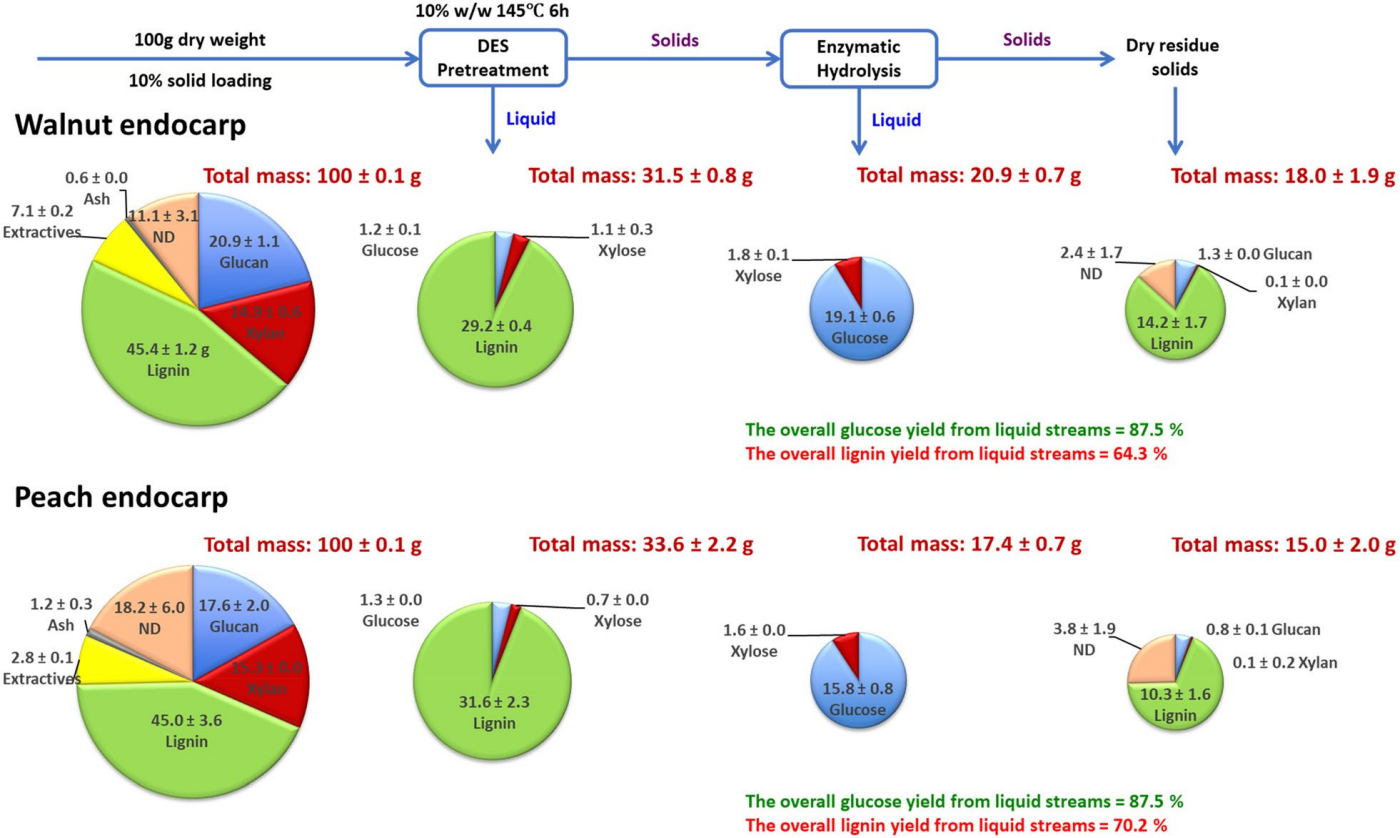
Mass flow of lignin, glucan, and xylan during DES pretreatment and enzymatic saccharification of walnut and peach endocarps. *ND* not detected. The mass reported only represents the counted fractions

The mass balances of the major components, glucan, xylan, and lignin for the DES pretreatment and enzymatic hydrolysis of walnut and peach endocarps are shown in Fig. 4. In general, a similar mass flow and allocation can be observed for both endocarps. Upon DES pretreatment, 40.5 and 34.2 g of pretreated solids were recovered for walnut and peach endocarps, respectively, based on 100 g dry untreated endocarp. The solid streams contain the majority of glucan, a portion of lignin and a slim of xylan. On the same basis, 29.2 g walnut endocarp lignin (64.3% of total) and 31.6 g peach endocarp lignin (70.2% of total) with a small amount of glucan and xylan went to the liquid fractions after pretreatment. Furthermore, approximately 19.1 g glucose and 1.8 g xylose from walnut endocarp and 15.8 g glucose and 1.6 g xylose from peach endocarp were recovered from the liquid streams of enzymatic hydrolysis. The overall yield of glucose from liquid stream were 87.5% and 87.5% for walnut and peach endocarps, respectively.

However, in comparison with the high overall glucan balance closure, mass balance for xylan was not well matched up. The overall balance closures of xylan were 17.2% for walnut endocarp and 13.3% for peach endocarp, respectively. Low xylose yield has been reported in a previous study using DES pretreatment of corncob [44]. Although it is challenging to compare results between various biomass types, DES solvent systems and operation conditions, we hypothesize that xylan underwent decomposition during DES pretreatment. To verify this hypothesis and better understand the reaction pathway of xylan, we introduced pure xylan as a model compound in DES under the same pretreatment condition and quantified the products recovered in the liquid fraction. As shown in Additional file 1: Table S1, only a slight portion of xylose (6.9%), can be detected in the pretreatment liquid. However, a total 37.6 wt% other products were recovered, including furfural, formic acid and levulinic acid; while 25.8 wt% of the starting material remained as solid residue. These preliminary results suggest that xylan was degraded during DES pretreatment; however future work is warranted to better understand the reaction kinetics and the impact of DES solvents on xylan degradation pathways and products.

It is worth noting that the costs of DES solvents are still higher than that of dilute acids and alkali, although DES solvents prove cheaper than many ILs [45]. Recovery and re-use of DES have been determined by previous studies [26, 30]. Considering differences in capital investment and operational costs on solvent separation, waste treatment and revenues of biofuels and lignin-derived products among difference pretreatment technologies, it is necessary to conduct a comprehensive techno-economic analysis of DES pretreatment process with respect to a biorefinery concept.

### Thermal properties of DES extracted lignins

**Fig. 5:**
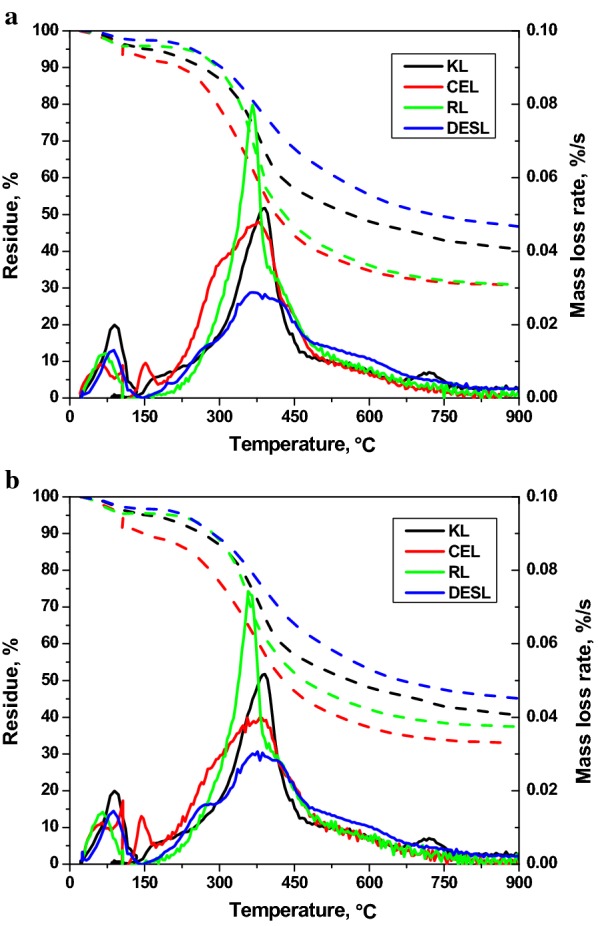
TG (solid lines) and DTG (dot lines) curves of Kraft lignin (KL), cellulolytic enzyme lignin (CEL), residue lignin (RL) and DES extracted lignin (DESL) from a) peach and b) walnut endocarps

The normalized thermogravimetric (TG) and differential thermogravimetric (DTG) curves of lignin samples, including Kraft lignin (KL), cellulolytic enzyme lignin (CEL), residual lignin in pretreated solid (RL) and DES extracted lignin (DESL) are shown in Fig. 5. Overall, continuous mass loss was observed over a wide temperature range and the first intense mass loss appeared between room temperature to 130 °C, which can be attributed to the evaporation of free and bound water in the lignin samples. The decomposition began around 150 °C and two major DTG phases can be observed from all lignin samples. The first phase appeared between 150 and 300 °C, which can be attributed to the decomposition of low molecular weight lignin polymers and the release of CO, CO_2_ and H_2_O from cleavage of the side chains of lignin molecules [46]. Following the first phase, the second phase, between 300 and 830 °C, showed the most intense peaks, indicating the release of volatiles from the degradation of large phenolic polymers.

Unlike CEL and DESL, RL demonstrated a different decomposition profile. The DTG curve commenced at 150 °C, rapidly rising to a maximal mass loss rate of 0.080 and 0.074%/s for peach and walnut RL, respectively. A slight mass loss rate shoulder was observed at approximately 400 °C before the peak finally finished at 830 °C. The significantly higher mass loss rate of RL before the shoulder when compared with CEL and DESL may be attributed to the impurities, including glucan, xylan and other un-determined contents; while the peak after the shoulder revealed the decomposition of lignin remained in RL [47, 48]. At the end of thermal degradation, the residual mass fraction followed an increase order of CEL < RL < KL < DESL. The significantly higher residue fraction of DESL than that of CEL and RL may be attributed to the arduousness of lignin decomposition due to the condensation during DES pretreatment, which can also explain why the DESL have a broader but lower DGT peak as compared to CEL and RL.

### Molecular weight distribution of DES extracted lignins

To better understand the lignin depolymerization process during DES pretreatment of endocarps, gel permeation chromatography (GPC) was applied to determine the molecular weight distribution. The weight average molecular weight (*M*_w_), number average molecular weight (*M*_n_) and polydispersity index (PDI) of the CEL, RL, and DESL are shown in Table 2. The molecular weights of CEL, representing the intact lignin in native plant, were significantly higher than that of RL and DESL, indicating that DES pretreatment is effective in depolymerizing the native lignin. The extent of size reduction was however less intense as compared to IL pretreated lignin with [C_2_C_1_Im][OAc] [49, 50]. It is possible that the depolymerized lignin partially repolymerized during DES pretreatment, which has been seen in a previous study on DES extracted sorghum lignin [51]. The PDI values reveal the heterogeneity of the size distribution of the lignin samples. The relative PDI value of RL was significantly higher than that of CEL and DESL for both peach and walnut endocarps. Results suggest that the CEL and DESL were more uniform in molecular weight than RL after DES pretreatment. The increasing in heterogeneity of the RL may be explained by either the simultaneous depolymerization and repolymerization of lignin oligomers during DES pretreatment or the uncompleted deconstruction due to inadequate contact.

**Table 2:** The number-average (*M*_n_) and weight-average (*M*_w_) molecular weights of Kraft lignin (KL) and cellulolytic enzyme lignin (CEL), residue lignin from solid residues after DES pretreatment (RL) and DES extracted lignin (DESL) from peach and walnut endocarps

	CEL			RL			DESL		
	*M*_w_ (g/mol)	*M*_n_ (g/mol)	PDI	*M*_w_ (g/mol)	*M*_n_ (g/mol)	PDI	*M*_w_ (g/mol)	*M*_n_ (g/mol)	PDI
Kraft	N/A	N/A	N/A	N/A	N/A	N/A	4952	2600	1.9
Peach	6129	2805	2.2	4780	1490	3.2	4344	2431	1.8
Walnut	7426	3551	2.1	4880	1616	3.0	4200	2460	1.8

*N/A* not applicable

### Structural and compositional characterization of DES extracted lignins

The FTIR spectra of CEL, RL and DESL of peach and walnut endocarps are shown in Additional file 1: Figure S3, to investigate the structural changes and chemical variations of DES pretreatment on endocarp lignins. All lignin samples exhibited a broad absorption band at 3400 cm^−1^, representing to the O–H stretching vibrations in phenolic and aliphatic O–H groups [52]; The intensity of this band decreased in DESL which can be attributed to the depolymerization and condensation reactions during DES pretreatment. The decreased peaks in DESL between 2920 and 2840 cm^−1^ represents CH_n_ bonds [53], suggesting the removal of alkane side chains. The bands at 1600, 1500 and 1420 cm^−1^ were attributed to aromatic ring stretch vibrations (C=C) and the C-H deformation bonding with aromatic ring vibration at 1450 cm^−1^ [54]. The increased peaks in DESL at 1220 and 1280 were corresponding to C–C, C–O and C=O stretching [55], which can be attributed to lignin condensation and side chains transfer. The bands at 1140 and 1120 cm^−1^ were associated with guaiacyl (S) and syringyl (G) units of lignin, respectively [55, 56]. The more intense band at ~ 1700 cm^−1^ in DESL than that of either CEL or RL suggested presence of more unconjugated C=O units. The significantly reduced S unit in DESL than CEL can be found in both peach and walnut endocarp, which were corresponding to the NMR analysis.

**Fig. 6:**
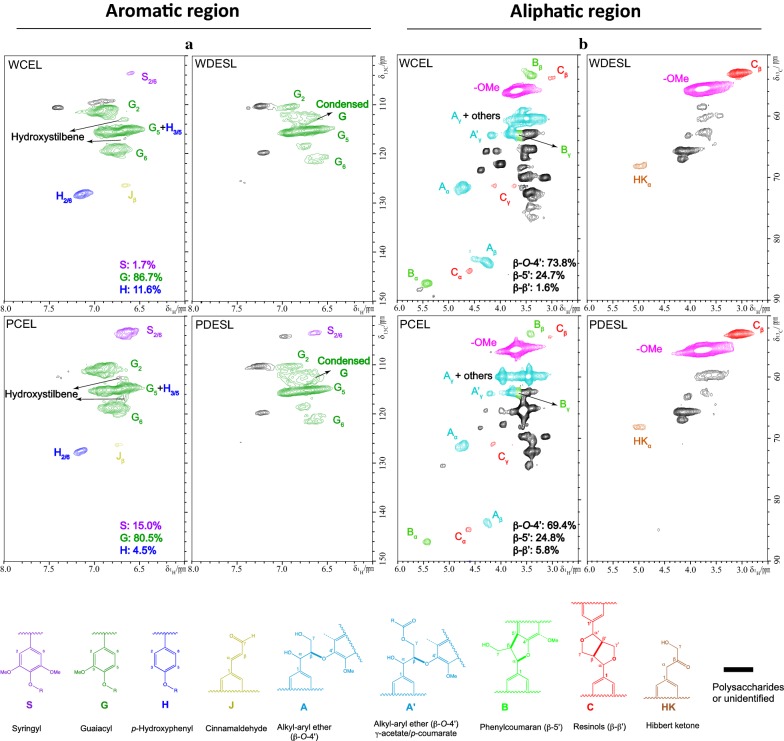
^13^C–^1^H (HSQC) spectra of aromatic regions (left) and aliphatic region (right) of walnut CEL (WCEL), walnut DES extracted lignin (WDESL), peach CEL (PCEL) and peach DES extracted lignin (PDESL). The structures of lignin compositional units and side-chain linkages were coded with colors corresponding to the cross peaks in the spectra

To examine the change in chemical structure of endocarp lignins through DES pretreatment, 2D ^1^H–^13^C HSQC NMR was applied to characterize the endocarp CELs and DES extracted lignins. The aromatic region (6.0–8.0/100–150 ppm) of the lignin samples, revealed key lignin monolignol subunits, as shown in Fig. 6a. 2D NMR spectra of aromatic regions showed that both walnut and peach CELs are SGH type lignin, and dominated by G unit accounting for up to 80% of all compositional units. The peach lignin had a much higher S unit (15.0%) than the walnut lignin (1.7%) and a lower H units (4.5% vs. 11.6%). The S/G ratios of peach and walnut CEL are 0.19 and 0.02, respectively. Hydroxystilbenes have been identified as important components of lignin in certain endocarps such as palm fruit, carnauba, and coconut [57, 58]. However, only trace level of signal that could corresponds to the hydroxystilbene in form of scirpusin structure has been observed in the peach and walnut lignin isolated in our study. After DES pretreatment, a large portion of S and all of H units were removed. In addition, a large amount of condensed G unit was observed after DES pretreatment, which can be explained by its high reactivity toward condensation during pretreatment [54]. The structural changes of lignin subunits in the endocarp lignins, i.e., removal of H and condensation of G units, are consistent with the results observed in the sorghum lignin treated with DES [51].

The aliphatic region (2.5–6.0/50–90 ppm) of the lignins, revealed the lignin inter-units and side chains, was shown in Fig. 6b. Both peach and walnut CELs were found to be dominated by β-*O*-4′ and β-5′ units accompanied with a minor amount of β-β′ linkages. After DES pretreatment, β-β′ linkages were significantly increased, at the expense of the removal of β-*O*-4′ and β-5′ linkages. The presence of Hibbert’s ketone (HK) (68.6/4.93 ppm) in DES lignin corroborates the cleavage of β-*O*-4′ linkages by DES. The results of side-chain linkages changes, i.e., substantial decreases of β-*O*-4′ and β-5′, increase of β–β′, and the formation of HK, in endocarp lignins are consistent with the DES treated sorghum lignin [51] and Douglas fir lignin [59]. The NMR spectra revealed that the endocarp lignins of peach and walnut undergo a similar structural change during DES treatment as other lignin species, such as sorghum and Douglas fir.

## Conclusions

Walnut and peach endocarps have high lignin content, bulk density, and energy density compared to other common biomass feedstocks, attributing to the unique plant cell wall structures. DES pretreatment was shown to be an effective method to fractionate endocarps in order to produce both sugar and lignin streams. More specifically, > 90% sugar yields were achieved during enzymatic hydrolysis of DES pretreated peach and walnut endocarps. Lignins were extracted at high yields of 64.3% for walnut and 70.2% for peach endocarps with more than 92% purity. Characterization of the recovered lignin streams demonstrated that DES pretreatment is effective in depolymerizing the native lignin while at the same time keeping thermal stability. The native walnut and peach CELs are SGH type lignin with dominant G units. The DES pretreatment significantly removes the S and H unit while condenses the G unit. Meanwhile, the relative abundance of β-β’ linkages in DES extracted lignin increased; nearly all β-*O*-4′ and a large portion of β-5′ linkages were removed during DES pretreatment.

## Methods

### Materials

The two endocarp feedstocks: peach pit (*Prunus persica*) and walnut shell (*Juglans nigra*) were collected in 2017 from Center for Crop Diversification at University of Kentucky. The remaining pericarp and mesocarp tissues were manually removed from the endocarps and the recovered endocarps were washed with DI water, and dried at 40 °C in a convection oven. Hybrid poplar (*Populus deltoides* × *P. nigra*, clone OP-367/433) and lodge pole pine (*Pinus contorta*), were obtained from the Idaho National Laboratory. The raw biomass feedstocks were grounded by a Wiley Mill to pass through a 20 mesh screen. Then the grounded biomass was sieved to acquire a particle size range of 0.25–0.425 mm. All chemicals and reagents were of analytical grade and purchased from Sigma-Aldrich (St. Louis, MO, USA) and Fisher Scientific (Waltham, MA, USA). Enzymes, cellulase (Cellic^®^ CTec2) and hemicellulase (Cellic^®^ HTec2) were provided by Novozymes North America (Franklinton, NC, USA).

### Compositional analysis

The percentage of biomass composition, including moisture, extractives, ash, glucan, xylan and lignin, was determined with a two-stage acid hydrolysis according to a NREL laboratory analytical procedure [60]. Following the two-stage acid hydrolysis, acid insoluble lignin was determined by the acid insoluble residue excluding the ash content. The quantity of acid soluble lignin was determined by UV–vis spectrometer at the absorbance of 205 nm. The amount of monomeric sugars, glucose and xylose, were measured by a Dionex Ultimate 3000 HPLC equipped with a refractive index detector and a Biorad Aminex HPX-87H column, using 5 mM H_2_SO_4_ as mobile phase at a flow rate of 0.4 ml/min and a column temperature set of 50 °C. Galactose, mannose, and arabinose contents were low or absent in raw biomass as analyzed by HPLC using Biorad Aminex HPX-87P column using water as mobile phase thus HPX-87H column was used for sugar analysis.

### Pretreatment

#### Deep eutectic solvent (DES) pretreatment and lignin recovery

The DES in the present study was synthesized from choline chloride and lactic acid with a molar ratio of 1:2. The eutectic mixture was prepared by mixing the two components in a beaker at their solid state, followed by heating the mixture in an oil bath at 60 °C with constant stirring until a homogeneous and transparent DES liquid (ChCl-Lac) was gained. For DES pretreatment, 2 g of endocarp biomass was slurried in an 18 g of DES, the endocarp biomass (10% biomass loading) was pretreated with the ChCl-Lac solvent in an ACE glass pressure vessel reactor at 145 ± 2 °C in an oil bath for 6 h [54]. The pretreatment was carried out with a constant stirring at 200 rpm. After pretreatment, the slurry was rinsed with 20 ml ethanol and centrifuged at 4000 rpm for 10 min to separate the pretreated solid and liquid fraction. Lignin was precipitated from the liquid fraction by adding water to the liquid until reaching a water: ethanol ratio of 1:9 [59]. Precipitated lignin was washed 5 times with a 1:9 ethanol/water solvent and the pretreated biomass was washed five time with ethanol to fully remove any remained DES solvent. And then the washed pretreated solids and lignin were freeze-dried for future use.

#### Dilute acid (DA) pretreatment

2 g of endocarp biomass was slurried in 18 g of 1% (w/w) sulfuric acid solution in a 20 ml SS316 stainless steel reactor and pretreated at 160 ± 2 °C in an oil bath for 40 min. After pretreatment, the slurry was centrifuged at 4000 rpm for 10 min to separate the solids and liquid. The recovered biomass solids were washed four times with 35 ml of hot DI water to remove any residual sugars and excess sulfuric acid and kept at 4 °C for further analysis.

#### Alkaline (AL) pretreatment

2 g of endocarp biomass was slurried in 18 g of 2% (w/w) NaOH and 0.5% H_2_O_2_ solution in a 20 ml SS316 stainless steel reactor and pretreated at 160 ± 2 °C in an oil bath for 60 min. After pretreatment, the slurry was centrifuged at 4000 rpm for 10 min to separate the solids and liquid. The recovered biomass solids were washed four times with 35 ml of hot DI water to remove any residual sugars and excess alkali and kept at 4 °C for further analysis.

### Enzymatic hydrolysis and mass balance

Enzymatic saccharification of untreated and pretreated endocarps were carried out according to the NREL laboratory analytical procedure [61]. The glucan loading used during enzyme hydrolysis was 1%. The cellulase (Cellic^®^ CTec2, protein content 188 mg/ml) was applied at enzyme loading of 20 mg CTec2 protein/g glucan supplemented with hemicellulase (Cellic^®^ HTec2, protein content 27 mg/ml) loading of 0.26 mg/g glucan. The saccharification was performed at 50 °C, 0.05 M citrate buffer and pH 4.8 in an orbital shaker. After 72 h of hydrolysis, the remaining solids were collected by centrifugation and washed four times with DI water to remove residual sugars, while the supernatant liquid fractions were analyzed by HPLC for the monosaccharides as mentioned in the composition analysis section. Mass balances (Glucan, xylan and lignin) were closed on the liquid and solid streams of fractionated endocarps after DES pretreatment and enzymatic hydrolysis on dry basis of 100 g starting biomass.

### Characterization of lignin and untreated and treated endocarps

#### Confocal laser scanning microscopy (CLSM)

Calcoflour White Stain (Sigma Aldrich, St Louis MO) was prepared by mixing Calcoflour White Stain with 10% Potassium Hydroxide at 1/1 (v/v) and specimens were soaked under the coverslip in solution for 1 min prior to imaging. Specimens were imaged under an Olympus FV1200 Laser Scanning Microscope at 60×. All images were captured using Fluoview software version 4.2 with the same settings: excitation wavelength of 405 nm, dichroic beam splitter of 405/488/559 nm, and a bright field range of 70 nm starting at 410 nm. Minimal processing was performed aside from fluorescence normalization. The figure was cropped and edited in Adobe Photoshop and Illustrator.

#### Staining and Imaging for Light Microscopy

A solution of Toluidine Blue was made by mixing 0.05% (w/v) Toluidine Blue (Sigma Aldrich, St Louis MO) with distilled water and a phloroglucinol stain was prepared fresh using 50% 1 M HCl and 50% distilled water with a 5% (w/v) of phloroglucinol. Biomass samples were briefly exposed to these solutions by immersing them between 3 and 5 min. Specimens were imaged under an Olympus stereomicroscope in bright field conditions. Images were captured using cellSens Dimension software (Olympus).

#### Scanning electron microscopy (SEM)

Images of the raw, pretreated endocarps and DES extracted lignin samples were obtained by a FEI Quanta 250 FEG SEM operating at SE mode under low vacuum (0.40–0.65 Torr). Samples were prepared for imaging by freeze-drying using an AdVantage 2.0 bench top lyophilizer (SP Scientific, Warminster, PA). The dried biomass samples were sputter-coated in gold and the imaging was performed at beam accelerating voltages of 2 kV.

#### Gel permeation chromatography (GPC) analysis

The samples were acetylated using acetic acid and acetyl bromide as published protocol for GPC analysis [62]. The weight-average molecular weight (*M*_w_) and number-average molecular weight (*M*_n_) of the lignin samples were determined by a Dionex Ultimate 3000 HPLC system, which equipped with a Mixed-D PLgel column (5 μm particle size, 300 mm × 7.5 mm i.d., linear molecular weight range of 200–400,000 u) and ultraviolet (UV) detector at wavelength of 280 nm.

### Fourier transform infrared spectroscopy (FTIR)

A Nicolet Nexus 870 FTIR was used to obtain FTIR spectra of the lignin samples. Spectra were obtained using an average of 64 scans between 400 and 4000 cm^−1^ with a spectral resolution of 2 cm^−1^. The raw FTIR spectra were baseline corrected and normalized using Omnic 6.1a software and compared in the range 700–2000 cm^−1^.

### Thermogravimetric analysis (TGA)

All TG and differential thermogravimetric (DTG) data were acquired using a Thermo Scientific Q500 TGA analyzer. In brief, 10 mg of lignin sample was placed in a crucible, heated in a nitrogen environment from room temperature to 105 °C ramping at 10 °C/min and held for 40 min to determine the moisture content. Then, temperature was increased to 900 °C ramping at 10 °C/min and held for 20 min to measure volatile content.

### Cellulolytic enzyme lignin (CEL)

The untreated endocarps were extracted with a mixture of toluene-to-ethanol ratio of 2:1 (v/v) [63, 64]. The extractives-free samples were grinded using a SPEX SamplePrep 8000D ball mill loaded with 10 × 10 mm balls at 550 RPM in a frequency of 5 min with 5 min pauses in-between for 1.5 h time in total. The milled fine powder was then subjected to enzymatic hydrolysis with a mixture (1:1 by volume) of Cellic^®^ CTec2 and HTec2 at 50 °C, 0.05 M citrate buffer and pH 4.8 in an orbital shaker for 48 h. The residue was isolated by centrifugation and enzymatic hydrolyzed one more time with fresh enzymes. The lignin-enriched residue was extracted with dioxane-water (96% v/v, 10.0 ml/g biomass) for 24 h. After separation of supernatant with residue, dioxane extraction was repeated one more time. The extracts were combined, roto-evaporated to reduce the volume at less than 45 °C and freeze dried. The obtained lignin samples, designated as CEL, was used for further analysis.

### Nuclear magnetic resonance (NMR) spectroscopic analysis

Two-dimensional heteronuclear single-quantum correlation NMR (2D HSQC NMR) spectroscopy of lignins were obtained at 25 °C on a Bruker Avance III HD 500-MHz spectrometer incorporated with a 5 mm N_2_ cryogenically cooled BBO H&F probe using Bruker pulse sequence (hsqcetgpspsi2.2). Test samples were prepared by dissolving 20 mg of CEL in 100 mg DMSO-*d*_*6*_ in a micro-NMR tube, while 40 mg of DES lignin in 0.5 ml DMSO-*d*_*6*_ in 5 mm NMR tube. The HSQC experiments were performed with the following acquisition parameters: spectra width 12 ppm in F2 (^1^H) dimension with 1024 data points (acquisition time 85.2 ms), 166 ppm in F1 (^13^C) dimension with 256 increments (acquisition time 6.1 ms), a 1.0-s delay, a ^*1*^*J*_C–H_ of 145 Hz, and 128 scans. The central DMSO-*d*_6_ solvent peak (δ_C_/δ_H_ at 39.5/2.49) was used for chemical shifts calibration. Assignment and the relative abundance of lignin compositional subunits and inter-unit linkage were estimated as described in literatures [63, 65]. For volume integration of monolignol compositions of syringyl (S), guaiacyl (G), and *p*-hydroxyphenyl (H), the cross peaks of S_2/6_, G_2_, and H_2/6_ contours were used with G_2_ integrals doubled. The C_α_ signals were used for volume integration for inter-unit linkages estimation. The abundances of aromatics and side-chain linkages were presented as percentage of total SGH units.

## Additional file

**Additional file 1.** Additional figures and tables.

## Abbreviations

DES: deep eutectic solvent
IL: ionic liquid
HBD: hydrogen-bond donor
HBA: hydrogen-bond acceptor
ChCl: choline chloride
EG: ethylene glycol
SEM: scanning electron microscopy
CLSM: confocal laser scanning microscopy
Lac: lactic acid
TG: thermogravimetric
DTG: differential thermogravimetric
KL: kraft lignin
CEL: cellulolytic enzyme lignin
RL: residue lignin from solid residues after DES pretreatment
DESL: DES extracted lignin
GPC: gel permeation chromatography
*M*_w_: weight average molecular weight
*M*_n_: number average molecular weight
