# Supplementary material for: Fractionation and characterization of lignin streams from unique high-lignin content endocarp feedstocks
Source: Biotechnol Biofuels. 2018 Nov 8;11:304. doi: 10.1186/s13068-018-1305-7 (PMC6222996; doi:10.1186/s13068-018-1305-7)
Supplement: Supplementary file 1 — Additional file 1. Additional figures and tables. [file 13068_2018_1305_MOESM1_ESM.docx]

***Additional Information***


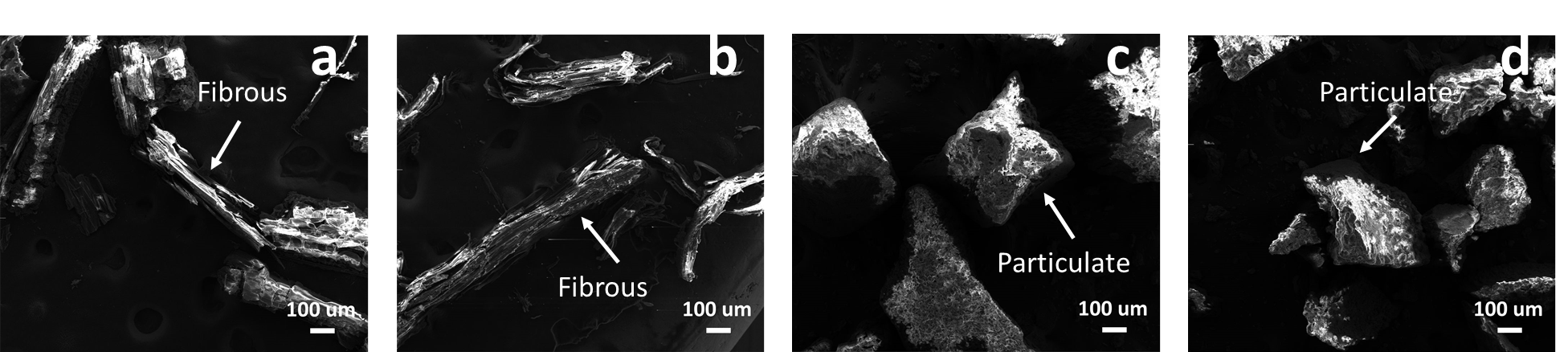


**Figure S1.** SEM images of raw biomass samples: **a)** switchgrass, **b)** pine wood, **c)** walnut endocarp, **d)** peach endocarp.


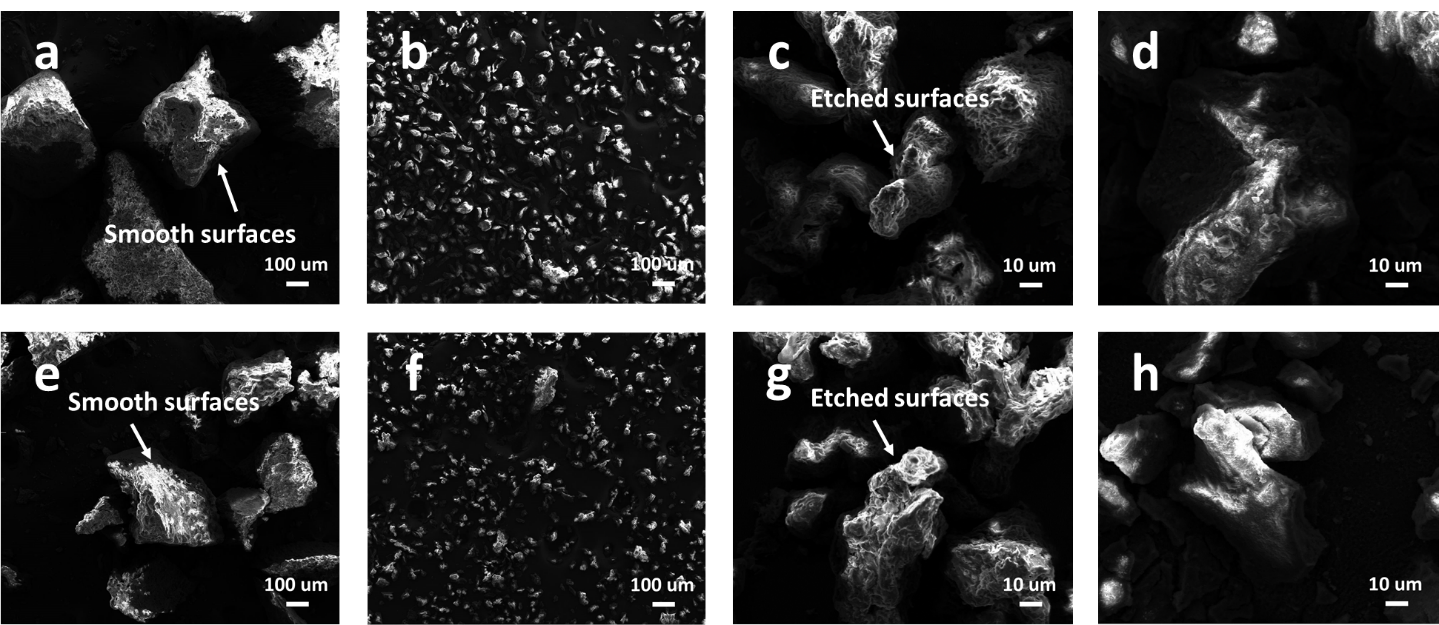


**Figure S2.** SEM images of unpretreated, DES pretreated and extracted lignin of walnut and peach endocarp: **a)** unpretreated walnut endocarp, **b)** and **c)** DES pretreated walnut solid, **d)** extracted walnut lignin, **e)** unpretreated peach endocarp, **f)** and **g)** DES pretreated peach solid, **h)** extracted peach lignin.

**2920**

**1280**

**1120**

**1140**

**1500**

**1600**

**3400**

**(a)**

**2920**

**1280**

**1500**

**1600**

**1120**

**1140**

**3400**

**(b)**

**Figure S3.** FTIR spectra of Kraft lignin (KL) and cellulolytic enzyme lignin (CEL), residue lignin (RL) and DES extracted lignin (DESL) from **a)** peach and **b)** walnut endocarps.

**Table S1.** Composition analysis for DES pretreatment of xylan and liquid fraction of endocarps*

|  | **Glucose, %** | **Xylose, %** | **Lignin,**  **%** | **Residue, %** | **5-HMF, %** | **Furfural, %** | **Formic Acid, %** | **Levulinic Acid, %** | **ND, %** |
| --- | --- | --- | --- | --- | --- | --- | --- | --- | --- |
| **Xylan** | ND | 6.9 ± 0.2 | ND | 25.8 ± 0.0 | 0.7 ± 0.0 | 33.9 ± 6.5 | 2.9 ± 0.1 | 0.2 ± 0.0 | 29.6 ± 6.8 |
| **Peach endocarp** | 0.0 ± 0.0 | 0.2 ± 0.0 | 31.6 ± 0.7 | 15.0 ± 0.8 | 0.2 ± 0.0 | 2.8 ± 0.1 | overlapped | overlapped | 16 ± 3.7 |
| **Walnut endocarp** | 0.1 ± 0.0 | 0.3 ± 0.1 | 29.2 ± 0.3 | 18.0 ± 1.3 | 0.4 ± 0.0 | 2.3 ± 0.0 | overlapped | overlapped | 9.2 ± 3.3 |

*Data represent means and errors are standard deviation from the mean of three independent replicates

**Table S2.** Pathway of each components in endocarps after DES pretreatment and enzymatic hydrolysis*

|  | **Peach** | | | **Walnut** | | |
| --- | --- | --- | --- | --- | --- | --- |
| **Content in, %** | **Glucan** | **Xylan** | **Lignin** | **Glucan** | **Xylan** | **Lignin** |
| **Raw endocarps** | 100 ± 11.4 | 100 ± 0.0 | 100 ± 8.0 | 100 ± 5.3 | 100 ± 4.0 | 100 ± 2.6 |
| **Lignin** | 6.7 ± 0.0 | 4.6 ± 0.0 | 70.2 ± 5.1 | 5.2 ± 0.4 | 7.6 ± 2.0 | 69.6 ± 0.9 |
| **Pretreated solids** | 80.9 ± 0.0 | 10.5 ± 0.0 | 0.0 ± 0.0 | 82.3 ± 2.6 | 12.1 ± 0.7 | 0.0 ± 0.0 |
| **Residue** | 4.1 ± 0.0 | 0.7 ± 1.3 | 22.9 ± 3.6 | 5.6 ± 0.0 | 0.7 ± 0.0 | 31.3 ± 3.7 |
| **Not determined** | 8.4 ± 11.9 | 84.3 ± 0.0 | 6.9 ± 16.7 | 6.9 ± 8.3 | 79.9 ± 6.7 | 0.0 ± 7.3 |

*Data represent means and errors are standard deviation from the mean of three independent replicates
